# TCSRNet: a lightweight tobacco leaf curing stage recognition network model

**DOI:** 10.3389/fpls.2024.1474731

**Published:** 2024-12-18

**Authors:** Panzhen Zhao, Songfeng Wang, Shijiang Duan, Aihua Wang, Lingfeng Meng, Yichong Hu

**Affiliations:** ^1^ Tobacco Research Institute of Chinese Academy of Agricultural Sciences, Qingdao, China; ^2^ Graduate School of Chinese Academy of Agricultural Sciences, Beijing, China; ^3^ Ji’an Tobacco Company of Jiangxi Province, Ji’an, China; ^4^ Jiangxi Branch of China National Tobacco Corporation, Nanchang, China

**Keywords:** tobacco leaf curing stage, image classification, lightweight network model, attention mechanism, smart agriculture

## Abstract

Due to the constraints of the tobacco leaf curing environment and computational resources, current image classification models struggle to balance recognition accuracy and computational efficiency, making practical deployment challenging. To address this issue, this study proposes the development of a lightweight classification network model for recognizing tobacco leaf curing stages (TCSRNet). Firstly, the model utilizes an Inception structure with parallel convolutional branches to capture features at different receptive fields, thereby better adapting to the appearance variations of tobacco leaves at different curing stages. Secondly, the incorporation of Ghost modules significantly reduces the model’s computational complexity and parameter count through parameter sharing, enabling efficient recognition of tobacco leaf curing stages. Lastly, the design of the Multi-scale Adaptive Attention Module (MAAM) enhances the model’s perception of key visual information in images, emphasizing distinctive features such as leaf texture and color, which further improves the model’s accuracy and robustness. On the constructed tobacco leaf curing stage dataset (with color images sized 224×224 pixels), TCSRNet achieves a classification accuracy of 90.35% with 158.136 MFLOPs and 1.749M parameters. Compared to models such as ResNet34, GhostNet, ShuffleNetV2×1.5, EfficientNet-b0, MobileViT-xs, MobileNetV2, MobileNetV3-large, and MobileNetV3-small, TCSRNet demonstrates superior performance in terms of accuracy, FLOPs, and parameter count. Furthermore, when evaluated on the public V2 Plant Seedlings dataset, TCSRNet maintains an impressive accuracy of 97.15% compared to other advanced network models. This research advances the development of lightweight models for recognizing tobacco leaf curing stages, providing theoretical support for smart tobacco curing technologies and injecting new momentum into the digital transformation of the tobacco industry.

## 1 Introduction

**Figure 1:**
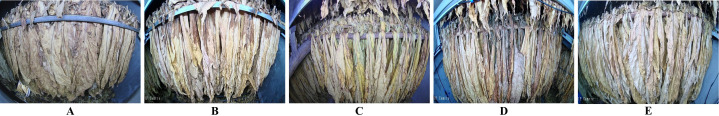
Tobacco leaf image dataset after curing. **(A)** Gray Tobacco **(B)** Mottled Tobacco **(C)** Undercooked Tobacco **(D)** Overcooked Tobacco **(E)** Standard Tobacco.

The tobacco industry plays a crucial role in the fiscal revenue of many developing countries, providing them with a substantial amount of tax income. This tax revenue has to some extent alleviated the financial pressure of these nations, enabling governments to invest in poverty alleviation, infrastructure development, and other critical areas. This has effectively promoted economic development and improved living standards in economically disadvantaged regions, making a positive contribution to achieving regional poverty reduction goals. Incomplete statistics show that China’s total tobacco output accounts for over 42% of the global total, and its cigarette production even makes up 32% of the world’s total, making it the largest tobacco producer and consumer globally (Liu, 2024). Among the key steps in tobacco processing, leaf curing is critical to ensuring cigarette quality. Even if the fresh tobacco leaves are of superior quality, their characteristics can be significantly degraded by improper curing, which in turn affects the economic benefits of the cured leaves (Li et al., 2021; Wang et al., 2019). Tobacco leaf curing refers to the process of removing moisture from fresh tobacco leaves under high-temperature conditions in curing barns or similar facilities, based on the growth characteristics of the leaves. By controlling appropriate curing parameters, such as temperature, humidity, and duration, the curing process regulates the enzymatic activities within the leaves, consolidating and developing the desirable attributes formed during leaf maturation. This transformation promotes the development of favorable leaf appearance and internal chemical composition, ultimately enhancing the overall quality of the cured tobacco leaves (Wang et al., 2009; Ding et al., 2023). Currently, the tobacco leaf curing process in China has developed into a refined and diversified set of intensive curing techniques across different regions. However, most curing methods are primarily based on 10 distinct stages characterized by observable changes in the appearance of the tobacco leaves. By monitoring alterations in color, texture, and moisture loss throughout the curing process, farmers can basically determine the current stage of the leaves and subsequently adjust the wet-bulb and dry-bulb temperatures, as well as the stabilization time in the curing barn, to complete the curing of the tobacco leaves. At this stage, the identification of curing stages in intensive curing barns still relies predominantly on manual assessment. Farmers observe the degree of yellowing and shrinking of the tobacco leaves through the barn windows to subjectively evaluate the curing status and adjust the curing process accordingly (Li et al., 2022b; Du et al., 2022). Nevertheless, the tobacco leaf curing process lasts for more than 150 hours, typically spanning 6 to 7 days, and the entire operation is highly subjective. This subjectivity can easily lead to mismatches between the curing stage and the curing techniques, resulting in an inability to guarantee the quality of the cured tobacco leaves (Sun et al., 2023). As shown in **Figure 1**, premature or delayed execution of the curing process can lead to the production of a significant amount of substandard tobacco, including gray, mottled, undercooked, and overcooked leaves, which severely impacts the quality of the cured tobacco. Therefore, researching methods for accurately identifying the stages of intensive tobacco curing, utilizing emerging image processing technologies for precise and automatic stage recognition, is a key focus in current tobacco curing research.

In recent years, machine vision technology and deep learning techniques have significantly advanced the development of smart agriculture, resulting in numerous high-quality research outcomes in the agricultural field. These advancements include real-time monitoring of crop growth and development (Arend et al., 2016; Ni et al., 2018), detection of crop diseases (Li et al., 2022a; Liu and Wang, 2020; Zeng et al., 2022; Zhang et al., 2023a; Zhou et al., 2024), and harvesting of fruits (Tang et al., 2020; Kang et al., 2020). In the tobacco sector, related research has primarily focused on diagnosing pests and diseases in tobacco fields (Chen et al., 2023), determining the maturity of tobacco leaves (Chen et al., 2021), and grading of cured tobacco leaves (Xin et al., 2023; Zhang and Zhang, 2011). Zhang et al. (2013) Utilize the HSI color space to perform color segmentation and recognition of tobacco leaves, and employ fuzzy logic to make decisions regarding the curing stage of the tobacco leaves. Wu et al. (2014) proposed the integration of color features from tobacco images with curing environment data, resulting in the development of a three-layer BP neural network regression prediction model to forecast the curing state of tobacco leaves. Wang et al. (2017) demonstrated that combining the color features and texture features of tobacco leaf images during the curing process as the input to a neural network can significantly improve the prediction accuracy. Wang and Qin (2021) proposed a method that utilizes tobacco leaf weight data for prediction, constructing a fusion model that incorporates the color and weight characteristics of tobacco leaves to forecast the curing status. Pei et al. (2024) utilized XGBoost to construct a model that integrates curing environment information and tobacco leaf image information to determine the curing stage of tobacco leaves. However, while the aforementioned research methods can enhance model performance by leveraging various information sources, they necessitate the manual design of feature engineering to address the fusion and weighting of heterogeneous information sources. The capacity for feature representation in images is influenced by subjective human factors, and most studies are limited to utilizing low-dimensional primary features such as color and texture, thereby failing to fully exploit the high-dimensional advanced feature information within images. In contrast, deep learning models possess the capability to automatically extract image features, process image information in real-time, and facilitate end-to-end deployment. This inherent ability allows deep learning approaches to more effectively harness the fusion of heterogeneous information sources, thereby improving model performance without the constraints imposed by manual feature engineering, which is often susceptible to subjective biases in feature representation. Jiang et al. (2023) utilized the existing Efficient Channel Attention (ECA) mechanism to replace the Squeeze-and-Excitation (SE) channel attention mechanism in EfficientNet, thereby achieving optimized discrimination of the tobacco leaf drying stage. Consequently, conventional machine learning techniques are unable to fully leverage the feature information within images, necessitating the fusion of multi-source information to maintain model accuracy. Furthermore, traditional Convolutional Neural Networks (CNNs) maintain high accuracy at the expense of computational resources, rendering them unsuitable for direct deployment in the resource-constrained environment of tobacco leaf drying houses.

Based on the aforementioned research, it is evident that deploying a tobacco leaf drying stage recognition model within a dense drying house requires the model to possess low computational complexity, a minimal number of parameters, and robust generalization capabilities. To address this, this study proposes a lightweight network model specifically designed for the recognition of the tobacco leaf drying stage—named TCSRNet. This model integrates an improved Inverted Residual Structure with the proposed MAAM. On the one hand, the enhanced Inverted Residual Structure and Ghost module facilitate the extraction of richer image features while significantly reducing the number of parameters and computational complexity within the model. On the other hand, the MAAM allows for the adaptive adjustment of feature importance based on the input data, accommodating the varying characteristics of different tobacco leaf drying stages and thereby enhancing the model’s robustness. This enables TCSRNet to effectively integrate features of varying levels and types within the resource-constrained environment of a drying house, allowing it to better capture the intricate feature information present in tobacco leaf images throughout the drying process. Consequently, the proposed lightweight network classification model, TCSRNet aligns with the practical conditions and environmental demands of intelligent tobacco drying, providing a theoretical foundation and technical support for the subsequent intelligent automation of dense drying processes.

In this paper, we propose a lightweight network model for tobacco leaf drying stage recognition, named TCSRNet. Our main contributions are as follows:

1. Improved Inverted Residual Structure: We incorporated the Inception structure to replace the standard convolution for channel expansion in the original Inverted Residual Structure, enabling the capture of tobacco leaf image features at different scales. Additionally, we leveraged Ghost convolution to reduce the image dimensionality, significantly decreasing the model complexity and parameter count.
2. Designed Multi-scale Adaptive Attention Module: This module employs diverse feature aggregation methods to extract versatile feature representations, and by computing the weighted combination of feature maps, it achieves adaptive focus on the important features within the tobacco leaf images, enhancing the model’s robustness.
3. Developed the TCSRNet Model: With a computational complexity of only 158.136M FLOPs and a parameter count of only 1.749M, the TCSRNet model achieves a recognition accuracy of 90.3% for the tobacco leaf drying stages, demonstrating superior performance compared to other deep learning network models.

## 2 Experimental materials

### 2.1 Image acquisition

**Figure 2:**
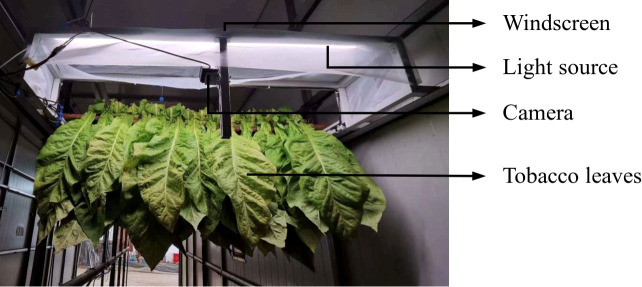
Tobacco leaf image acquisition device.

The experiments were conducted in 2023 in Anfu County, Jiangxi Province, Luonan County, Shaanxi Province, and Xichang City, Liangshan Prefecture, Sichuan Province. The tested variety was the locally grown Yunyan 87. The fresh tobacco leaves were produced using the local standardized tobacco production and management practices, ensuring uniform leaf quality. The experiments used biomass curing barns. The local promotion technology was used for curing. An intelligent curing image acquisition device designed by the Tobacco Research Institute of the Chinese Academy of Agricultural Sciences was used to collect tobacco leaf images, as shown in **Figure 2**. The device uses an LT-P4A50-C industrial high-temperature camera and a 25 W standard photography light source. A 200-mesh windscreen is installed above the device. The industrial camera is fixed on a stainless-steel frame, level with the tobacco sticks and tilted 45 degrees downward, with the light source facing the tobacco leaves and evenly spaced 15 cm apart. An image is captured every 5 minutes and uploaded to the server.

### 2.2 Image enhancement

**Figure 3:**
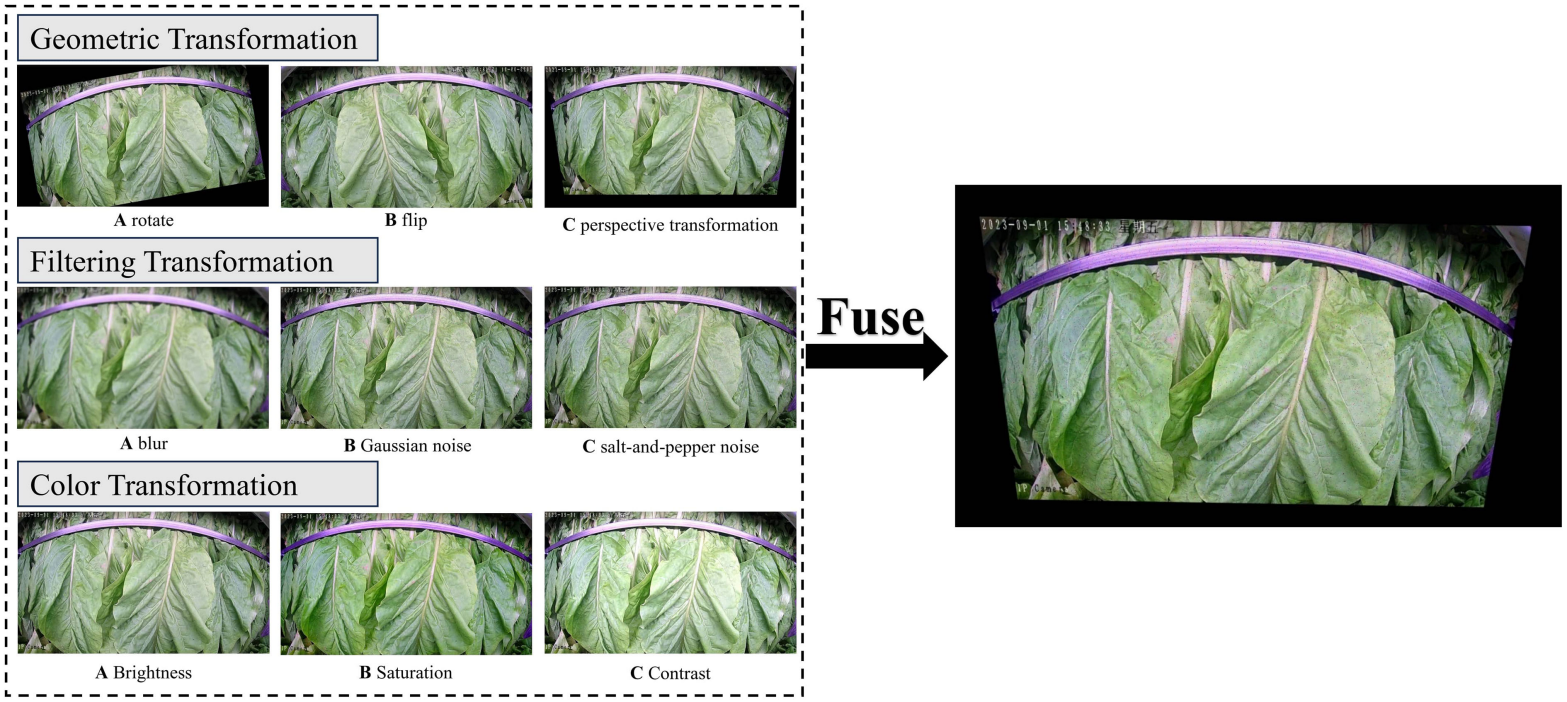
Tobacco leaf image enhancement.

This study aims to simulate the image acquisition process in different curing barns under real-world conditions. To address issues related to camera positioning and angle offsets during installation in various curing barns, geometric transformations were applied to the images. Additionally, to mitigate the effects of water vapor, dust, and fine particulate matter generated by the high-temperature and high-humidity environment during the curing process, filtering techniques were employed. Color transformations were also conducted to address the variations in luster and color disturbances presented by the light sources and different qualities of tobacco leaves. Furthermore, to prevent the imbalance of category samples for typical tobacco curing stages and to ensure that small sample sizes can be effectively trained, additional data augmentation methods, such as scaling, were utilized to increase the dataset’s sample size and avoid overfitting. As illustrated in **Figure 3**, a variety of enhancement techniques were applied to the tobacco leaf images, including varying degrees of geometric transformations (such as affine transformations, rotations, and flips), filtering processes (including noise and blur reduction), and color transformations (such as adjustments to brightness, saturation, and contrast). The objective was to simulate the numerous challenges faced when acquiring tobacco leaf images in real environments and to apply multiple enhancement methods in an overlapping manner, rather than relying on a single approach. This multifaceted enhancement strategy enables the model to learn more complex features during the curing stages in diverse environments, thereby improving the robustness and generalization performance of the model’s classification capabilities.

### 2.3 Dataset construction

Throughout the curing process, the tobacco leaves undergo changes, transitioning from the yellowing stage, to the color-fixing stage, and finally to the drying stage. Based on the reference to the three-stage curing process (Yang et al., 1995), the five-stage and five-corresponding curing process (Gao et al., 2002), and the eight-point curing process (Xu et al., 2012), and in consultation with professional curing experts, the industry standard (as shown in **Table 1**) was adopted, with a focus on the appearance and color of the leaves, as well as the critical temperature points. The entire dataset of images collected during the curing process was then divided into 10 typical tobacco leaf curing stages.

**Table 1:** Classification standards for tobacco leaf drying stages.

Curing Stage	Dry Bulb Temperature	Wet Bulb Temperature	Degree of Yellowing	Degree of Drying	Tobacco Leaf Image
1	36	35	Leaf margins turn yellow; leaves yellow approximately 30%	Leaves are soft	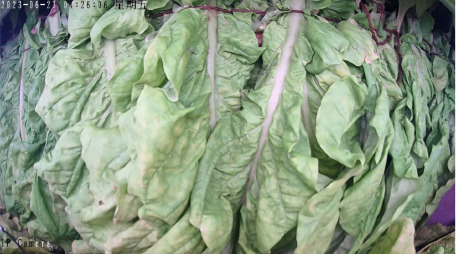
2	38	36	Leaves yellow approximately 70%	Main veins soften; slight tip curling	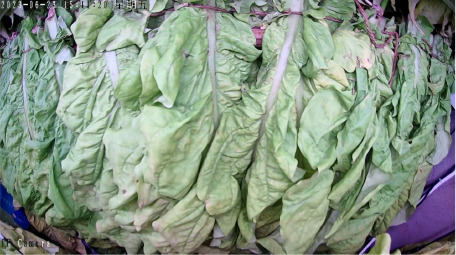
3	40	37	Leaves are nearly completely yellow	Tips curled and edges rolled; slight tip curling at the base	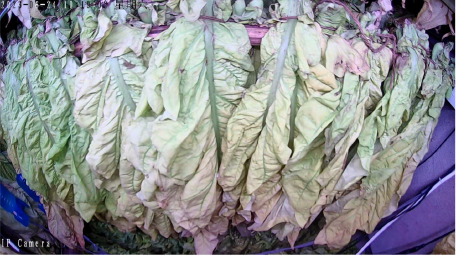
4	42	37	Leaf color is yellow with white; main veins entirely white and shiny; over two-thirds of lateral veins turned white; tips are dry and scorched	Leaves are approximately 30% dry	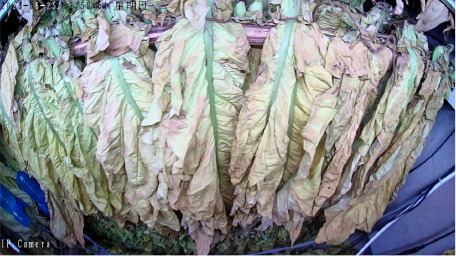
5	44	37	Yellow veins with green stripes	Leaves are approximately 50% dry	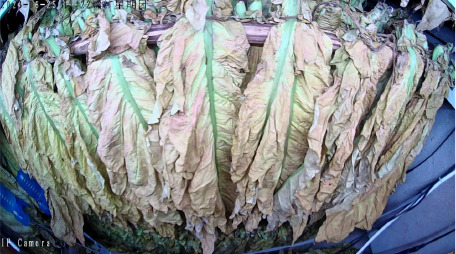
6	46	38	Yellow veins with yellow stripes	Leaves are approximately 70% dry	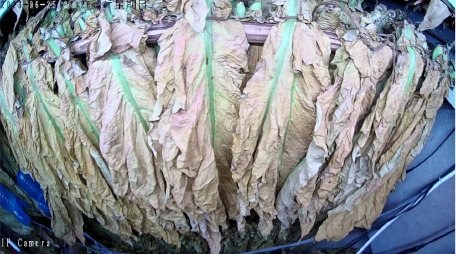
7	50	38	Main veins turn white	Leaves are approximately 90% dry	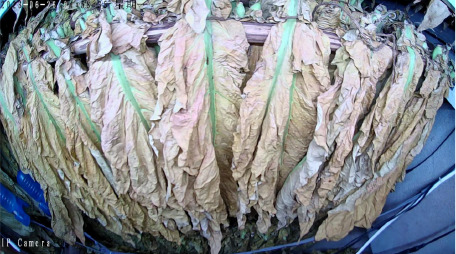
8	54	38	/	Leaves are mostly dry; main veins slightly white	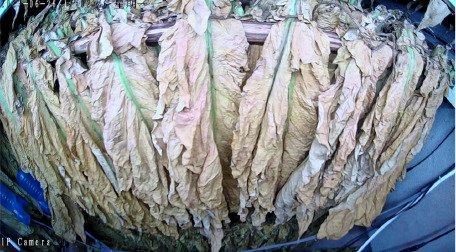
9	60	40	/	Main veins are approximately 50% dry	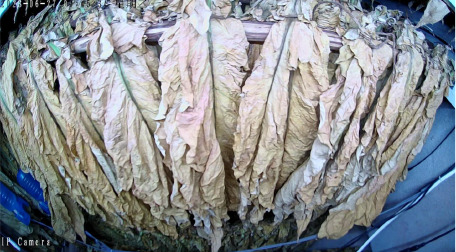
10	68	42	/	Main veins are completely dry	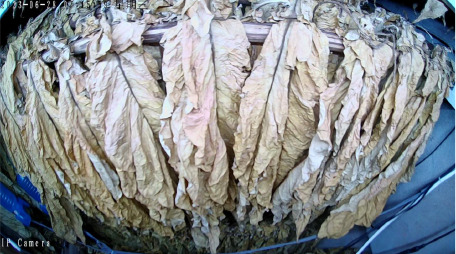

From the three locations of Anfu, Jiangxi, Luonan, Shaanxi, and Xichang, Sichuan, 3 curing cycles from each location, totaling 9 curing cycles, were selected as the training dataset. One curing cycle from each location, totaling 3 curing cycles, was used as the validation dataset, and another 3 curing cycles, one from each location, were used as the test dataset. Data augmentation was only applied to the training dataset, resulting in 48,164 augmented training images, 6,039 validation images, and 5,986 test images. To facilitate subsequent processing, the image size was reduced to 224×224 pixels using Python 3.11.0.

## 3 Model design

### 3.1 Model architecture

**Figure 4:**
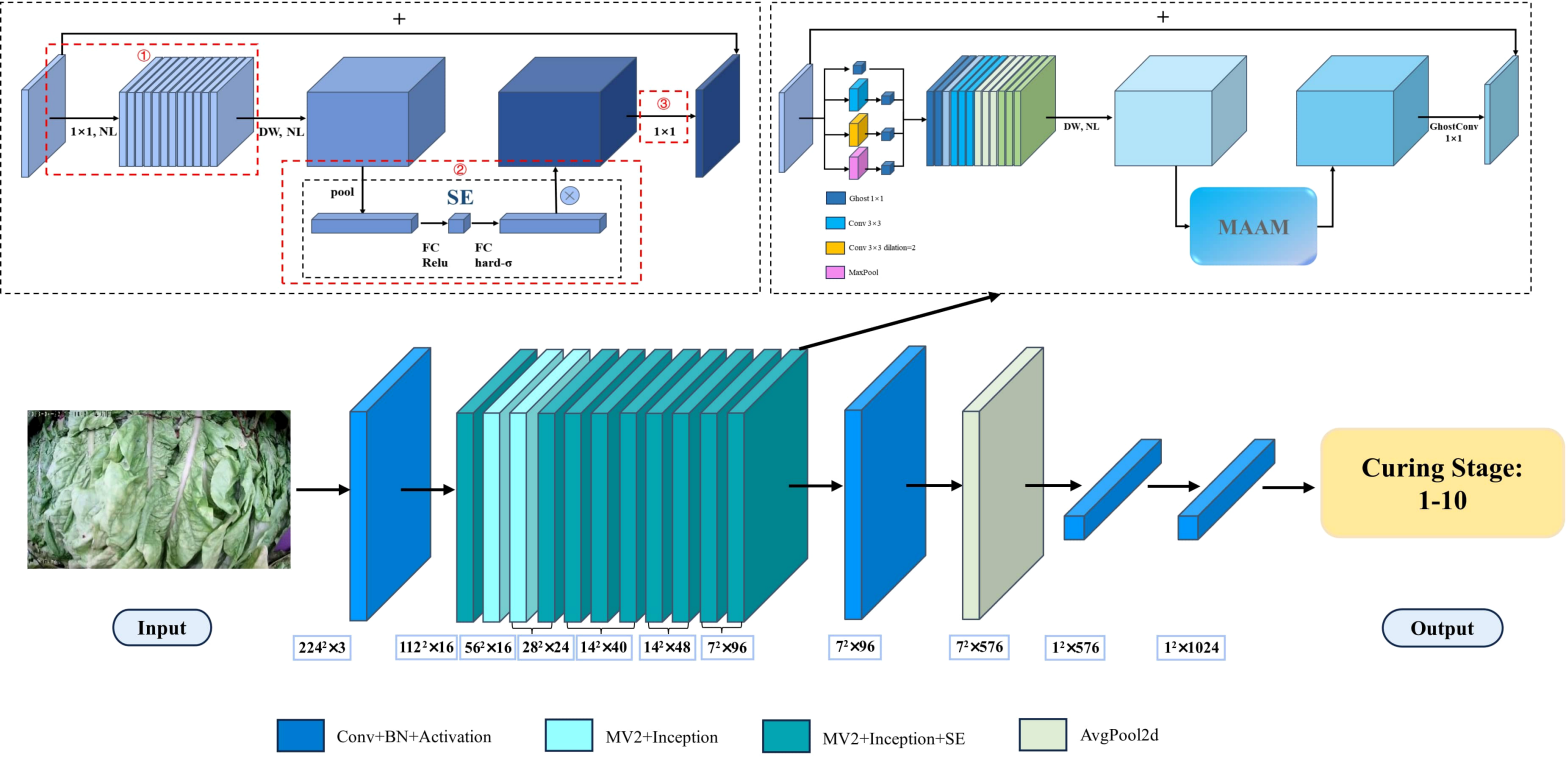
The structure of tobacco leaf curing stage recognition net.

Due to the constraints of computational resources within the curing barns, the deployment of complex models is not feasible. Therefore, this study adopts the lightweight MobileNetV3 model as the backbone network structure. Howard et al. (2019) proposed that MobileNetV3 is based on the foundations of MobileNetV1 (Howard et al., 2017) and MobileNetV2 (Sandler et al., 2018), inheriting the depth-separable convolutions from V1 and the linear bottleneck residual structure from V2. The model has been further updated, with improvements made to the building blocks, the use of NAS-searched parameters, and a redesign of the time-consuming layers. This has resulted in not only an increase in image classification accuracy but also an improvement in inference speed. As shown in **Figure 4**, the research has made improvements to the original MobileNetV3 architecture, including the Inverted Residual Structure and the attention module, to design a lightweight tobacco leaf curing stage recognition model, TCSRNet. Firstly, an Inception-like structure is used to replace the initial 1×1 expansion convolution layer in the Inverted Residual Block, allowing for the extraction of multi-scale tobacco leaf features through different branch convolution and pooling operations. Secondly, Ghost convolutions are employed to fuse the multi-scale features and perform dimensionality reduction in the final bottleneck layer, reducing the model complexity and improving its generalization capability. Lastly, a novel attention module, MAAM, is designed to effectively enhance the model’s perception and representation capabilities of the input data, thereby improving the overall performance of the model.

### 3.2 Improved inverted residual structure

#### 3.2.1 Inception structure

**Figure 5:**
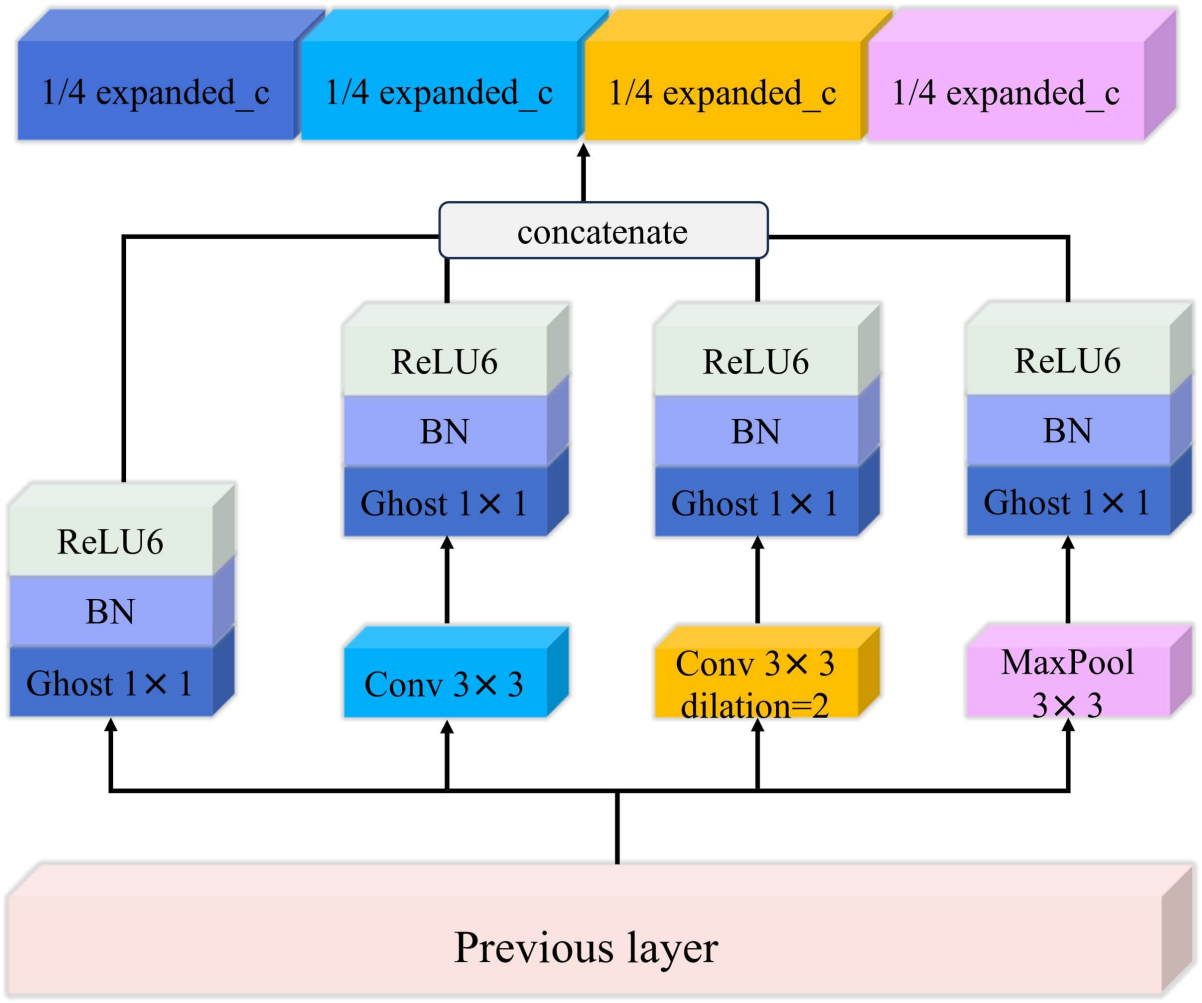
The structure of inception.

The research has made improvements to the original Inverted Residual Structure by incorporating an Inception structure to extract multi-scale features, which enhances the model’s adaptability to various types of input data. The parallel extraction of multi-scale feature information improves the model’s recognition capabilities. The Inception module, proposed by Google’s Szegedy et al. (2016), is based on the core idea of combining different convolution layers in a parallel manner. The result matrices from the various convolution layers are concatenated along the depth dimension, forming a deeper matrix that aggregates visual information at different scales, facilitating the extraction of features at multiple scales. Compared to the standard 1x1 convolution-based expansion operation in the original Inverted Residual Module, the use of the Inception structure offers several advantages. As shown in **Figure 5**, firstly, the Inception structure, with its parallel operations of 1x1 convolution, 3x3 convolution, 3x3 dilated convolution (dilation rate = 2), and max-pooling, better adapts to different types of input data and extracts multi-scale image feature information in parallel, enhancing the model’s expressive capability for the input data. Secondly, the use of 1x1 Ghost convolution to fuse and expand the multi-scale features allows for the comprehensive utilization of the different feature information extracted by the various branches, effectively reducing the model complexity and mitigating the overfitting issue. Lastly, each parallel branch includes normalization and activation functions, further improving the non-linear expression capability of the Inverted Residual Structure and enhancing the model’s generalization ability.

#### 3.2.2 Ghost module

**Figure 6:**
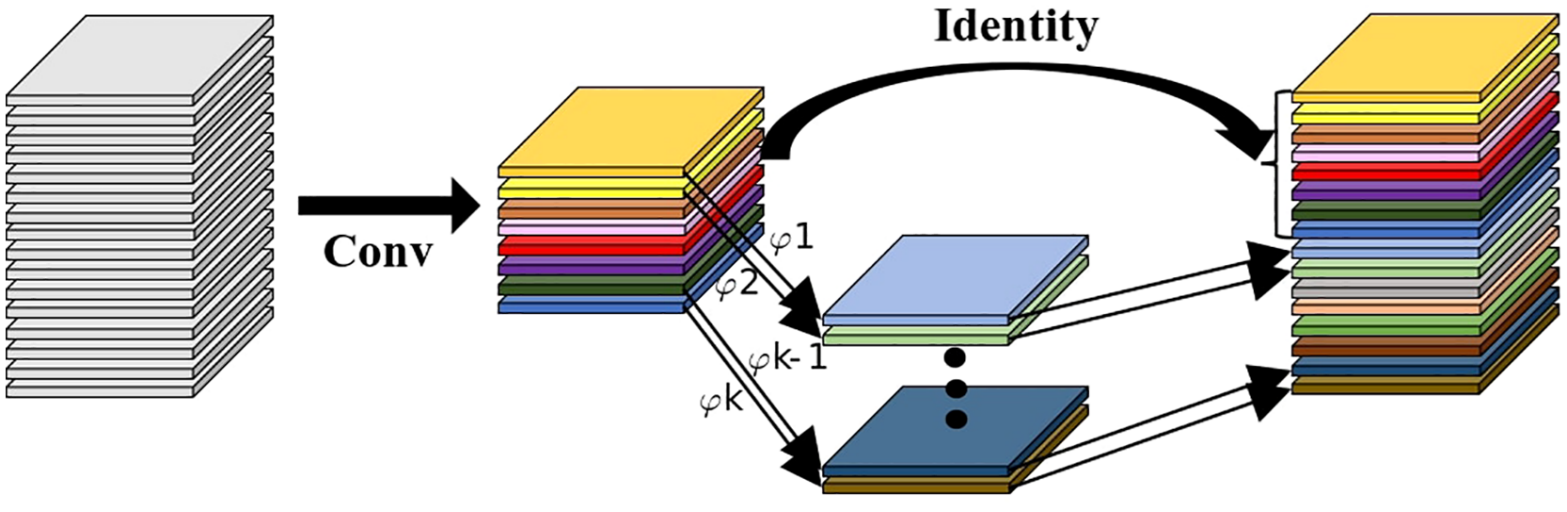
The structure of GhostNet module.


Due to the limited computational resources in the tobacco curing barns, the incorporation of the Inception structure to extract multi-scale information would increase computational complexity and parameter count. The introduction of Ghost convolution can mitigate these issues by reducing both computational complexity and parameter count, thereby enhancing the model’s inference speed and facilitating its practical deployment. The Ghost module, proposed by Han et al. (2020), was designed to address the redundancy of feature maps in deep neural networks. As shown in **Figure 6**, this module generates additional feature maps through a series of linear operations, creating a cost-effective means of producing rich information while ensuring model accuracy and reducing the number of parameters. Assuming the generation of *n* channel feature maps, a scaling factor of *s*, a convolution kernel size of *k*, and an input data channel count of *c*, the parameter compression ratio when using the Ghost module can be calculated as shown in Equation 1, allowing the network’s parameters to be compressed to $\frac{1}{s}$ of the original count. In the TCSRNet network, Ghost convolution is primarily applied within the multi-branch Inception structure and the final dimensionality reduction layer of the Inverted Residual Block. By introducing randomly generated low-dimensional “ghost” filters in the convolution layers, the model’s parameter count can be effectively reduced while maintaining performance, thereby minimizing the risk of overfitting and ensuring the operational efficiency of the lightweight model.

(1) $$r_{c}=\frac{n\cdot c\cdot k\cdot k}{\frac{n}{s}\cdot k\cdot k+(s-1)\cdot \frac{n}{s}\cdot k\cdot k}$$

#### 3.2.3 Multi-aggregation attention module

**Figure 7:**
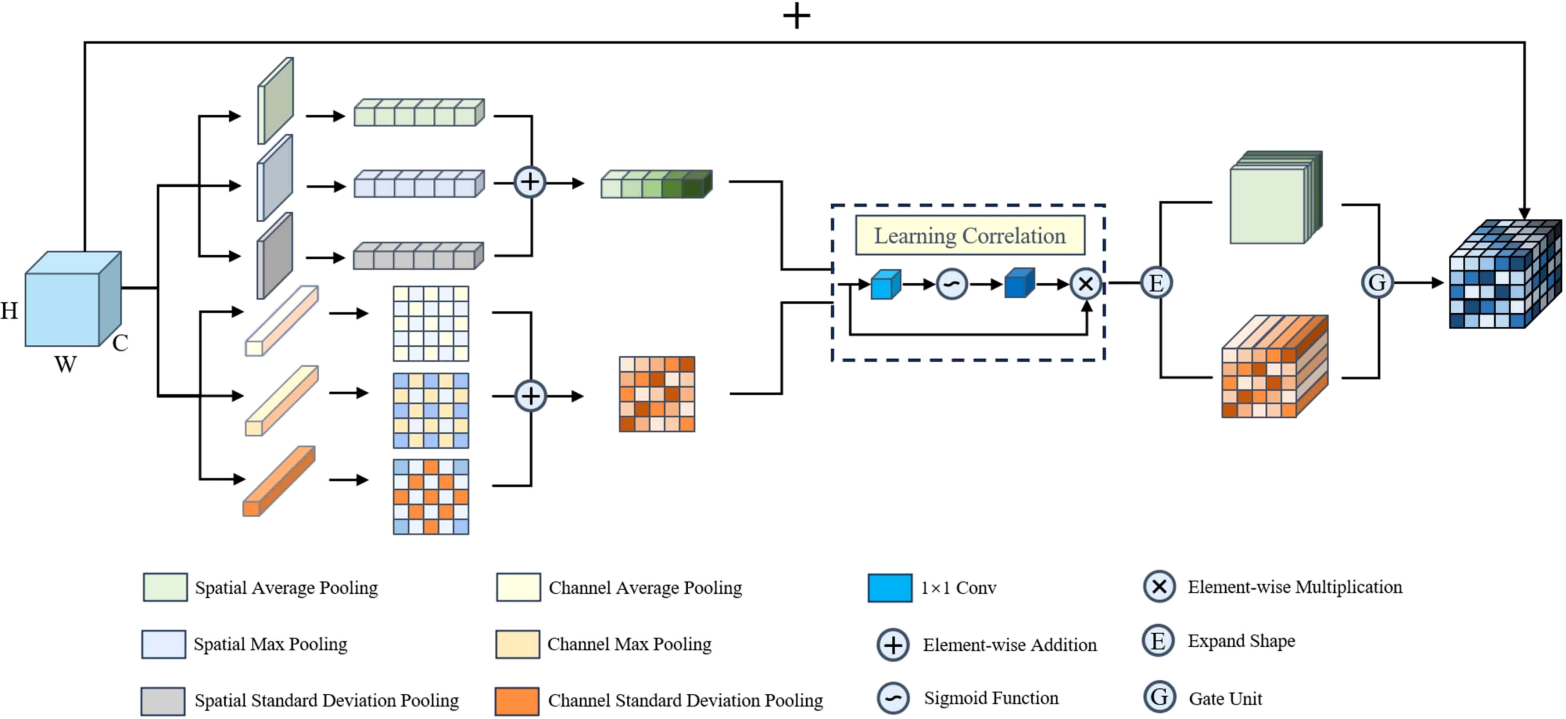
Multi-Aggregation attention module.

Although CNNs are remarkably powerful in image representation, they exhibit deficiencies in expressing spatial information. Therefore, in the original version of MobileNetV3, the SE attention module was introduced and placed in the middle of the bottleneck layer. Hu et al. (2020) used two fully connected layers and an activation function to provide updated weight values. However, the SE attention module only considers the interdependencies between channels, neglecting positional information. Given that the color, shape, and texture of tobacco leaves undergo non-linear changes during the curing process, we propose the MAAM. As shown in **Figure 7**, we first apply different pooling operations, such as average pooling, max pooling, and standard deviation pooling, to the input feature maps along the channel and spatial dimensions. This allows us to capture the multi-scale semantic information of the input features from different statistical perspectives, providing a more comprehensive representation of the tobacco leaf’s state. Next, we elementwise add the different-scale semantic information and use 1x1 convolution and a sigmoid function to adjust the importance of channel and spatial information, effectively highlighting the crucial channels or spatial locations. Finally, we employ a gating unit to control the feature fusion, dynamically adjusting the weights of the channel and spatial information in the final output. This enhances the model’s perception and robustness in tobacco leaf curing state assessment, and also strengthens its real-time response capability in complex environments, providing strong support for the practical application of intelligent curing monitoring systems. Compared to other attention mechanism modules, the MAAM has several key advantages:

**Multi-scale Feature Extraction:** By applying various pooling operations, such as average, max, and standard deviation pooling, to the channel and spatial dimensions of the input feature maps, we comprehensively describe the semantic information of the input features from different statistical perspectives. This consideration of both channel and spatial dimensions allows the model to gain a deeper understanding of the complex changes in tobacco leaves during the curing process.

**Highlighting Feature Importance:** After extracting multi-scale feature information, we use elementwise addition to fuse these features. This method effectively preserves the relative relationships between different scales, allowing the features at each scale to complement each other and form a richer semantic representation. Additionally, the flexibility of 1x1 convolution and the sigmoid function enables the model to adapt to different feature distributions, thus more effectively highlighting the important visual information.

**Effective Feature Fusion:** The introduction of a gating unit allows the model to dynamically evaluate the importance of channel and spatial features in the input information, and automatically adjust their weights based on the specific input data. This dynamic weight adjustment mechanism ensures that the model can always focus on the most influential features for the task, thereby improving overall performance. Compared to traditional simple weighted summation or concatenation operations, the gating mechanism is more flexible in handling the mutual relationships and interference between features, enhancing the model’s robustness in complex environments.

## 4 Results and analysis

### 4.1 Experimental setup

The experiments were conducted on a Windows 11 operating system, using Python 3.9.5 and the PyTorch 2.0.0 deep learning framework. The CUDA version used was 11.8, and the hardware configuration included a 16 vCPU Intel(R) Xeon(R) Platinum 8352V CPU @ 2.10GHz, 24 GB of memory, and an NVIDIA GeForce RTX 4090 GPU.

### 4.2 Classification metrics

TP represents the number of correctly classified positive samples, TN represents the number of correctly classified negative samples, FN represents the number of false negative samples, and FP represents the number of false positive samples. To accurately and comprehensively describe the data processing performance, the following metrics were used for evaluation: Accuracy, Precision, Recall, F1-Score, and Specificity.


(2) $$\text{Accuracy}=\frac{\text{TP}+\text{TN}}{\text{TP}+\text{TN}+\text{FP}+\text{FN}}$$

(3) $$\text{Precision}=\frac{\text{TP}}{\text{TP}+\text{FP}}$$

(4) $$\text{Recall}=\frac{\text{TP}}{\text{TP}+\text{FN}}$$

(5) $$\text{F}1-\text{Score}=\frac{2\cdot \left(\text{Precision}\cdot \text{Recall}\right)}{\left(\text{Precision}+\text{Recall}\right)}$$

(6) $$\text{Specificity}=\frac{\text{TN}}{\text{TN}+\text{FP}}$$

Image classification efficiency is evaluated using two metrics: Floating Point Operations (FLOPs) and the number of parameters. FLOPs can be used to measure the model complexity, while the number of parameters represents the weights and biases that need to be learned in the model. Fewer FLOPs and parameters mean that the model requires less storage space, less memory during inference, and fewer computational resources during training. This is an important consideration for practical deployment, as it allows the model to be efficiently implemented on resource-constrained devices, such as those used in the tobacco curing process. By optimizing the model’s FLOPs and parameter count, the overall efficiency and feasibility of the system can be significantly improved, making it more suitable for real-world applications.

### 4.3 Model training

**Figure 8:**
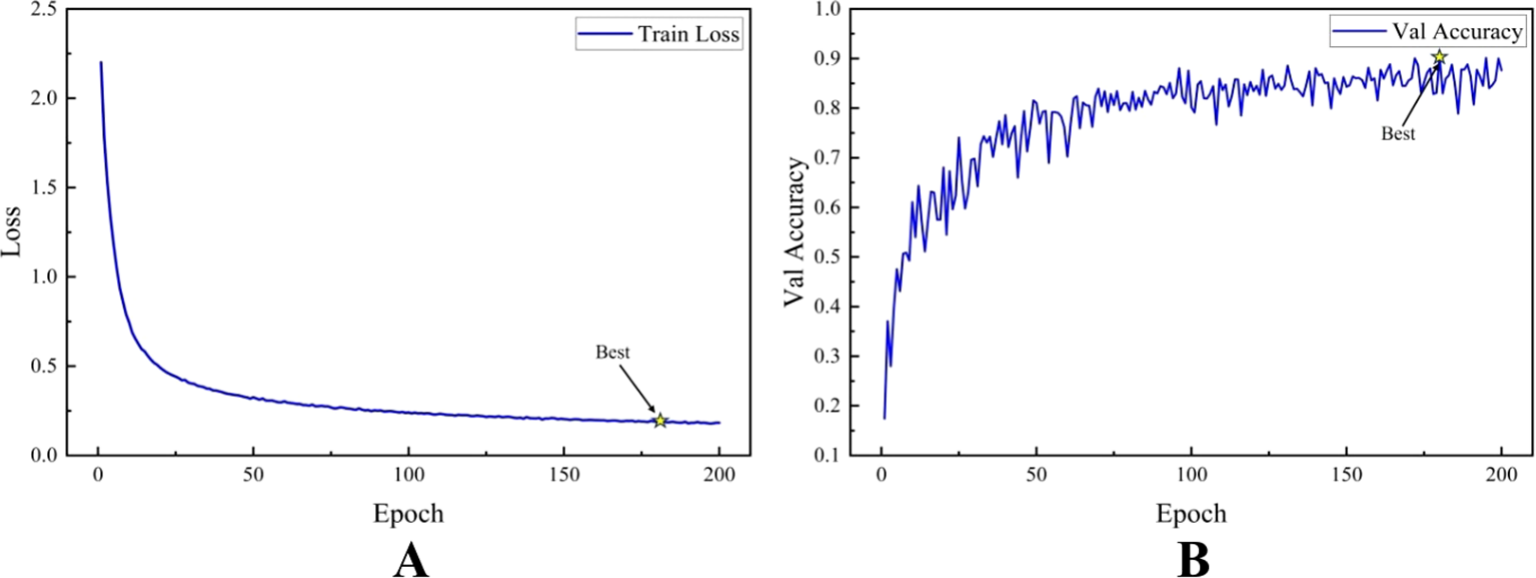
Schematic diagram of TCSRNet training process. **(A)** Variation of training set loss value **(B)** Variation of validation set accuracy.

**Figure 8** shows the trends of the training loss and validation accuracy of the TCSRNet during the training process. As observed from the figure, the training loss function value decreases rapidly as the number of training epochs increases, and then gradually stabilizes. This indicates that the model quickly learned effective feature representations in the early stages of training, leading to a rapid improvement in its predictive capabilities. In the later stages of training, the loss function value further decreases slowly, stabilizing around 0.2 after 100 epochs. We selected the model weights corresponding to the highest validation accuracy during training as our output, and applied this model to the test set for evaluation. This approach ensures that the final model achieves the best performance on the validation data, which is representative of the true data distribution, and can therefore be expected to generalize well to the test set.

### 4.4 Ablation experiment

To validate the feasibility and effectiveness of the designed TCSRNet model, we conducted an ablation study using MobileNetV3-Small as the baseline model. The ablation study involved evaluating the impact of the Inception structure, Ghost module, and MAAM on the model’s performance. The results are presented in **Table 2**.

**Table 2:** Ablation experiments.

Normal Convolution	Inception	Ghost	SE	MAAM	Accuracy
√			√		87.8
		√	√		87.4
		√		√	88.3
	√	√	√		89.6
	√	√		√	90.3

The original MobileNetV3-Small network, which uses standard convolution and SE attention mechanism, achieved an accuracy of 87.8%. The Ghost convolution, which generates “primary” features using fewer standard convolution kernels and then uses linear transformation to generate “ghost” features, reduces the overall computational complexity and the number of parameters. When the standard convolution in the baseline model was replaced with Ghost convolution, the accuracy was 87.4%. Although this resulted in a slight sacrifice in accuracy, it significantly reduced the model’s computational cost. The SE module primarily focuses on global features, and may not be effective in capturing the importance of small objects in the image, thus failing to improve the recognition performance of small objects. In contrast, the MAAM combines average pooling, max pooling, and standard deviation pooling, allowing the model to comprehensively capture the diverse statistical characteristics of the input features and extract richer feature representations. Replacing the SE module with MAAM resulted in an accuracy of 88.3%. To simultaneously capture both local and global features, improve computational efficiency, and enhance the overall recognition performance, the Inception structure was introduced in the tobacco leaf curing stage recognition model. The Inception structure can effectively extract multi-scale features, enhancing the model’s expressive capabilities, and achieved an accuracy of 90.3%. In summary, the Inception structure helps the model better capture local features (textures, spots) and global features (shapes, structures), the Ghost module significantly reduces the model’s inference overhead while minimizing the sacrifice in accuracy, and the MAAM attention, composed of efficient pooling operations and simple weighted summation, contributes to the improvement of the model’s expressive capabilities through diverse feature representations.

### 4.5 Comparison of different attention mechanisms

To intuitively demonstrate the impact of different attention mechanisms, we computed and compared the effects of various attention mechanisms (ECA, MSCA, CBAM, CCA, SimAM, MAAM) on model accuracy and other performance metrics within the improved residual structure framework.

**Figure 9:**
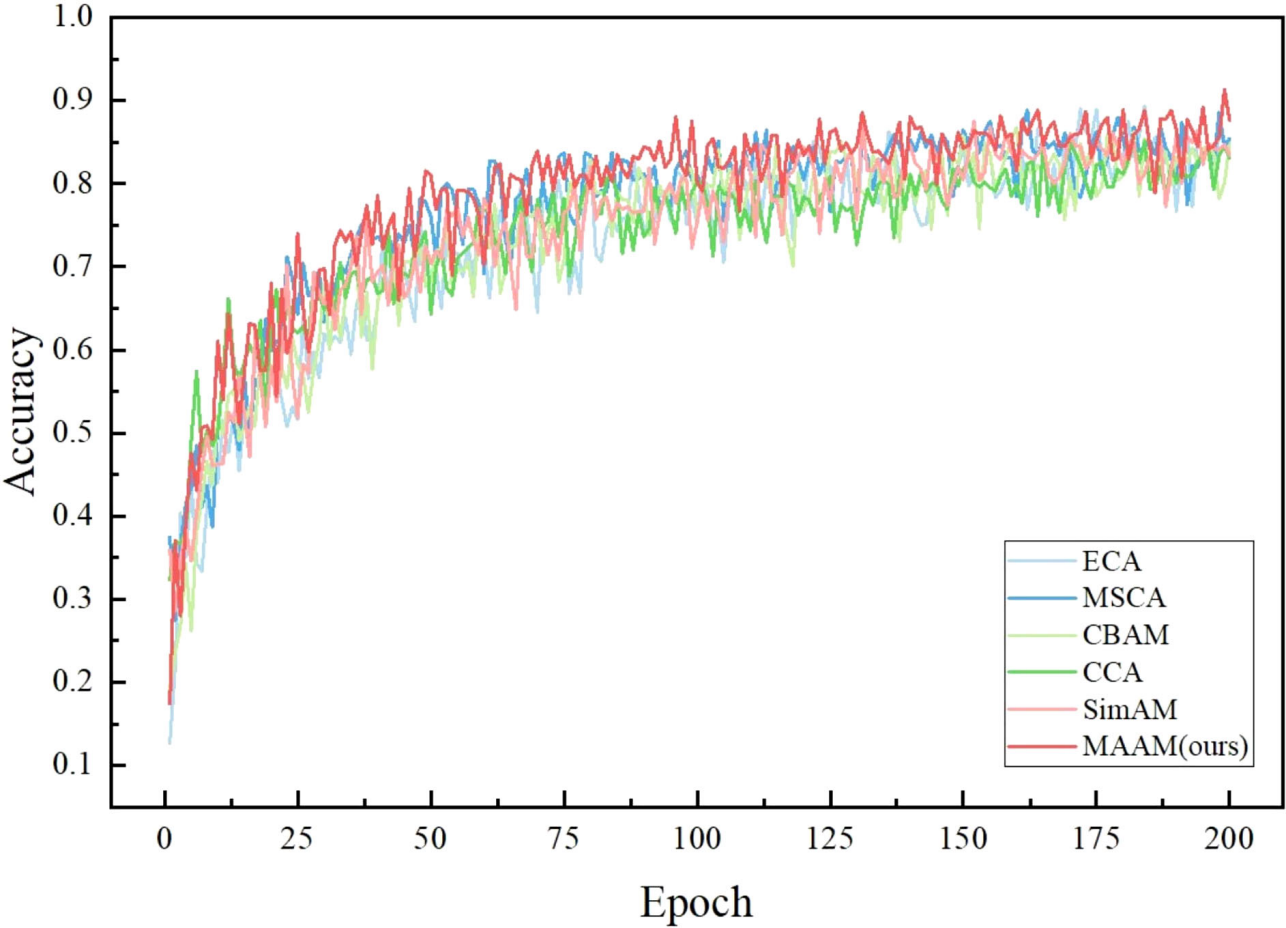
Schematic diagram of accuracy variation for different attention mechanisms.

As shown in **Figure 9**, we can clearly observe the numerous advantages of the MAAM model during the training process compared to other attention mechanism models. First, the accuracy curve of MAAM quickly reaches a high level at an early Epoch stage. This phenomenon indicates that MAAM can learn effective feature representations more rapidly, enabling the model to quickly focus on critical information within the input data and accelerating the model’s convergence process. Secondly, the MAAM accuracy curve demonstrates high stability with minimal fluctuations, a characteristic that highlights the model’s stability during training. This means that the MAAM attention mechanism enhances the model’s robustness, significantly reducing the risk of dramatic performance variations. Finally, MAAM maintains an accuracy rate higher than other models throughout the entire training process, with particularly outstanding performance in the later Epochs. This result further validates MAAM’s highly efficient capability in capturing and utilizing key features from input data, thereby improving the overall model performance.

As shown in **Table 3**, MAAM also demonstrates significant advantages in the comparison of performance metrics. First, MAAM ranks among the top models with an accuracy of 0.9033, while the accuracy of other attention mechanisms ranges from 0.8505 to 0.8934, highlighting MAAM’s superiority in feature capturing and information integration. The multiple attention mechanisms employed by MAAM provide a distinct advantage in effectively integrating and utilizing input information, significantly enhancing the overall performance of the model.Secondly, in terms of computational efficiency, MAAM achieves a frames per second (FPS) rate of 0.3004, slightly lower than CBAM’s 0.3122. MAAM’s latency is 88.0553 milliseconds, only better than CCA’s 135.0216 milliseconds. While other attention mechanisms have certain advantages in latency, their accuracy is significantly lower than that of MAAM. This indicates that MAAM successfully achieves high accuracy while maintaining reasonable inference speed, demonstrating its strong competitiveness in real-time applications.Furthermore, regarding resource usage, although the model sizes of ECA and SimAM are 3.9638 MB and 3.9636 MB, respectively, with a memory usage of 24.2890 KB, which allows them to excel in terms of resource consumption, they consequently sacrifice accuracy and FPS. In contrast, MAAM has a model size of 6.6730 MB and memory usage of 28.8388 KB, showcasing superior resource efficiency.

**Table 3:** Performance metrics of different attention mechanisms.

Model	Accuracy	FPS	Latency (ms)	Model Size (MB)	Memory Usage (KB)
CBAM	0.8676	0.3122	55.4707	5.6956	30.3740
CCA	0.8505	0.2957	135.0216	8.2962	33.0517
ECA	0.8934	0.2834	78.1321	3.9638	24.2890
MSCA	0.8886	0.2893	84.7065	8.3931	26.1875
SimAM	0.8766	0.2960	81.5219	3.9636	24.2890
MAAM	0.9033	0.3004	88.0553	6.6730	28.8388

In summary, the design philosophy of MAAM embodies a novel multi-attention mechanism that effectively captures key features of input data, allowing the model to demonstrate efficiency and stability during training and inference. Compared to other models, MAAM not only leads significantly in accuracy but also maintains a good balance in computational efficiency and resource usage. This multidimensional optimization enhances MAAM’s application potential in complex tasks, particularly in real-time and resource-constrained scenarios, where MAAM exhibits greater adaptability and practicality.

### 4.6 Comparison of different models

To further validate the effectiveness of the proposed TCSRNet model, we introduced and compared different models on the same dataset. These include heavyweight network models such as ResNet50, as well as lightweight network models like GhostNet, ShuffleNetV2×1.5, EfficientNet-b0, MobileViT-xs, MobileNetV2, MobileNetV3 large, and MobileNetV3 small, which are advanced classification models. The comparison models were all tested on their original model and parameter settings frameworks, and the experimental results were obtained on the test set.

As shown in **Table 4**, compared to the heavyweight network model ResNet50 and the lightweight network models GhostNet, ShuffleNetV2×1.5, EfficientNet-b0, MobileViT-xs, MobileNetV2, MobileNetV3 large, and MobileNetV3 small, the TCSRNet model has achieved significant performance improvements. In the task of recognizing the tobacco leaf drying stage, the TCSRNet model proposed in this study achieved an accuracy of 90.3%. Compared to the other lightweight networks GhostNet, ShuffleNetV2×1.5, EfficientNet-b0, MobileViT-xs, MobileNetV2, and MobileNetV3 small, the increase was 2.90%, 0.97%, 0.68%, 4.74%, 1.56%, and 3.28% respectively. In terms of Precision, the TCSRNet model improved by 0.60% to 5.00% compared to the other models on the dataset. In terms of Recall, the TCSRNet model improved by 0.40% to 6.42% compared to the other models on the dataset. In terms of F1-Score, the TCSRNet model’s F1 score was 0.10% to 5.20% higher than the other models. In terms of computational complexity, it is only 3% of ResNet50, and in terms of the number of parameters, it is only 7% of ResNet50, which is lower than the other models to varying degrees.

**Table 4:** Comparative analysis of classification metrics for different models.

Model	Accuracy	Precision	Recall	F1-Score	Specificity	FLOPs/M	Parameter/M
ResNet50	0.9112	0.897	0.896	0.895	0.990	4132	23.529
GhostNet	0.8745	0.861	0.861	0.855	0.986	196.618	4.215
ShuffleNetV2×1.5	0.8938	0.891	0.892	0.888	0.988	305.816	2.489
EfficientNet-b0	0.8967	0.887	0.895	0.888	0.989	411.562	4.020
MobileViT-xs	0.8561	0.847	0.845	0.842	0.984	743.485	1.937
MobileNetV2	0.8879	0.886	0.867	0.870	0.987	326.284	2.237
MobileNetV3large	0.9038	0.898	0.906	0.899	0.989	232.970	4.215
MobileNetV3small	0.8783	0.864	0.860	0.859	0.987	61.177	1.528
TCSRNet	0.9035	0.897	0.899	0.896	0.989	158.136	1.749

### 4.7 Confusion matrix

To more intuitively and comprehensively demonstrate the classification accuracy of different models in the tobacco leaf curing stage, we have created a confusion matrix. This allows us to clearly understand the correct and incorrect classifications of the model for each category, thereby evaluating the overall performance of the model and helping to fully grasp the model’s classification capabilities.

**Figure 10:**
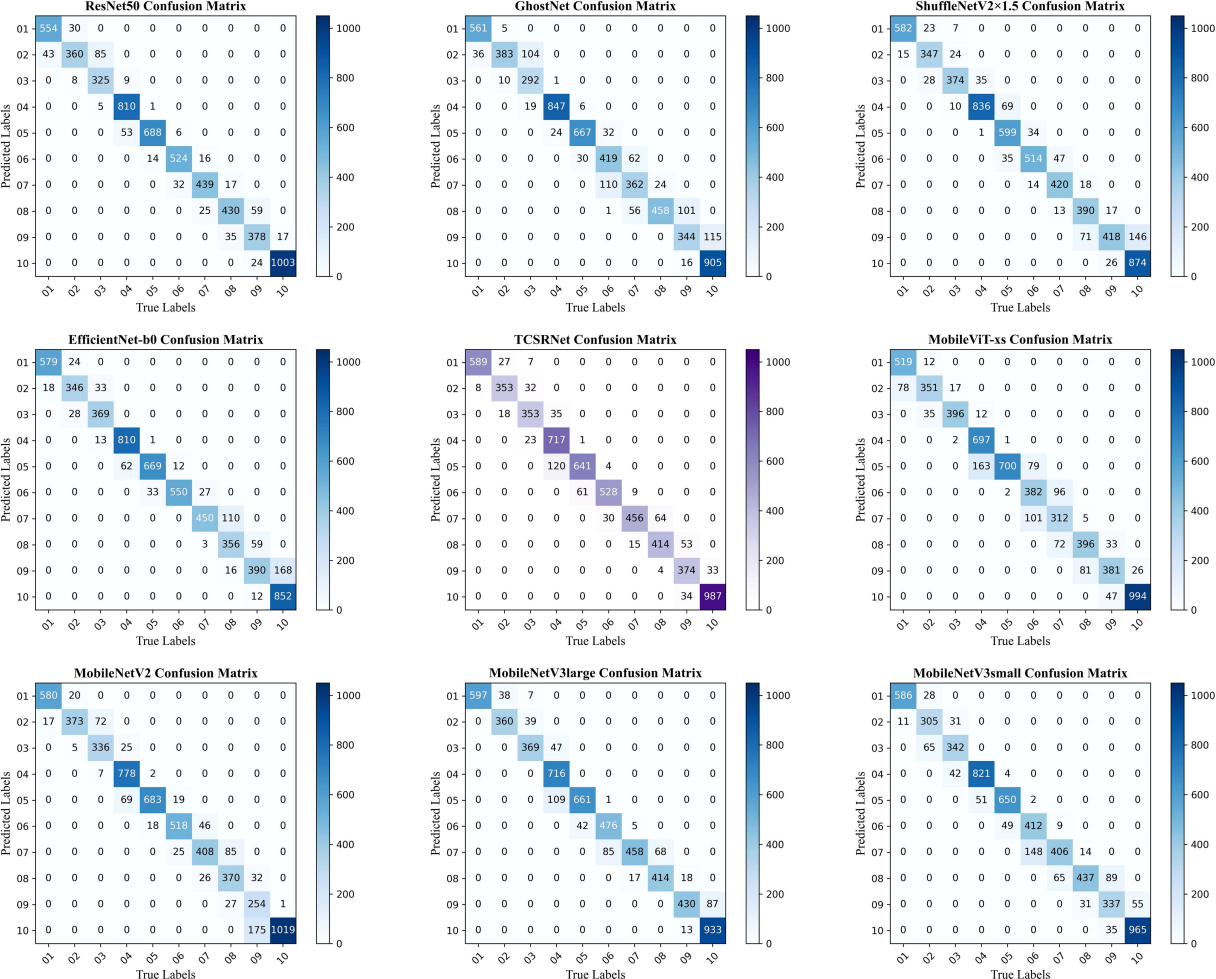
Schematic diagram of confusion matrices for different models.

As shown in **Figure 10**, the errors of each model are predominantly concentrated in stages 7 to 10, which correspond to the latter phase of the color-setting process and the curing stage of the tobacco leaf. This phenomenon may be attributed to the relatively minor changes in characteristics during these stages, where the color tends to become uniform, exhibiting a similar orange-yellow hue. Such visual similarity significantly complicates the task of distinguishing between the tobacco curing stages 7 to 10 based solely on visual assessment. Furthermore, during this period, the moisture content of the tobacco leaves is generally quite low, with the entire leaf, except for the main vein, approaching a state of dryness. This similarity in moisture conditions further exacerbates the complexity of feature differentiation, presenting additional challenges for the model in its recognition tasks.

### 4.8 Public dataset

To validate the performance of our enhanced model, we applied it to the widely utilized V2 plant seedling dataset. The dataset was divided into training, validation, and test sets in a ratio of 6:1:3. It encompasses 3 types of plants (common wheat, maize, and sugar beet) and 9 varieties of weeds (black-grass, common chickweed, cleavers, scentless mayweed, small-flowered cranesbill, shepherd’s purse, loose silky-bent, charlock, and fat hen). Through comparative analysis, as presented in **Table 5**, our enhanced model achieved an impressive accuracy of 97.06% in the plant seedling recognition task, surpassing the performance of other models. This outcome substantiates the model’s effectiveness and superiority.

**Table 5:** Accuracy of TCSRNet and other advanced models on the V2 plant seedling dataset.

Proposed	Model	Test Set Accuracy (%)
(Mu et al., 2022)	Faster R-CNN-FPN	95.61
(Rahman et al., 2020)	ResNet50	96.21
(Makanapura et al., 2022)	EfficientNetB0	96.52
(Zhang et al., 2023b)	Improved MobileNetV1	96.63
Ours	TCSRNet	97.06

## 5 Results and analysis

Currently, the identification of tobacco leaf drying stages in dense curing barns primarily relies on manual assessment based on visual observations of changes in leaf color, texture, and other characteristics, which introduces a significant degree of subjectivity. Moreover, most existing models prioritize accuracy at the expense of computational efficiency, making practical deployment in resource-constrained curing environments challenging. In response to these issues, this paper proposes a lightweight model for tobacco leaf drying stage recognition, designed to leverage limited computational resources to enhance the model’s ability to extract multi-scale features from images of drying stages while simultaneously reducing model complexity and parameter count to improve generalization capability. This research addresses two primary concerns: (1) reducing model complexity and parameter count while ensuring recognition accuracy, and (2) conducting a comparative analysis with existing lightweight classification networks to demonstrate the feasibility of the proposed model.

To tackle the first issue, the model incorporates innovative techniques such as the Inception structure, Ghost convolution, and MAAM. The Inception structure effectively extracts multi-scale features, enhancing the model’s sensitivity to visual information of varying granularity and improving its ability to capture visual characteristics such as texture and color of tobacco leaves. Ghost convolution significantly reduces the model’s computational complexity and parameter count through parameter sharing while maintaining robust feature extraction capabilities, thereby facilitating deployment in environments with limited computational resources. MAAM adaptively enhances the model’s perception of critical visual information, improving feature discrimination and bolstering generalization performance in complex curing conditions.

For the second issue, a comparative analysis is conducted between TCSRNet and classical classification networks. Experiments on the tobacco leaf image dataset will evaluate the model based on metrics such as accuracy, precision, recall, F1 score, FLOPs, and parameter count. A comprehensive analysis will demonstrate the feasibility of the proposed model relative to existing classification networks.

Despite the progress made in this study, there are still two main challenges to address. First, the heterogeneity of the tobacco datasets and regional differences may lead to significant variations in model performance across different datasets. For instance, the same model may achieve higher classification accuracy during the tobacco curing stage when applied to data from the same region; however, its accuracy could decrease noticeably when applied to data from different regions. The performance discrepancies across datasets should not be directly used as a measure of algorithm effectiveness, but rather reflect the method’s suitability for specific application contexts. Therefore, it is essential to conduct thorough testing and validation across different datasets in practical applications to ensure the selected algorithm’s applicability and robustness in various environments. Secondly, this study primarily evaluates the algorithm’s computational complexity by comparing FLOPs and the number of model parameters, but it has not conducted systematic testing in actual embedded systems or industrial environments. Although a comprehensive analysis of FLOPs and parameter count has been performed in this study, and the model demonstrates good computational performance, its real-world performance still requires further validation, particularly in practical scenarios where hardware resources are limited or environmental interference is present. Future research will focus on experimental validation on embedded platforms, particularly assessing the algorithm’s adaptability and performance in application environments with constrained computational resources or stringent real-time requirements, such as in tobacco curing rooms.

## 6 Conclusion

In this study, we developed a lightweight model, TCSRNet, for the recognition of tobacco leaf drying stages in dense curing barns. By integrating the Inception structure, Ghost convolution, and MAAM, the model effectively balances recognition accuracy, computational efficiency, and generalization performance. Comparative experiments on the tobacco leaf drying stage dataset showed that TCSRNet achieved an accuracy of 90.3%, with a computational complexity of 158.136M FLOPs and a parameter count of 1.749M, outperforming other lightweight classification networks. This provides a reliable technical solution for real-time automated monitoring and quality assessment in complex curing environments.

## Data Availability

The raw data supporting the conclusions of this article will be made available by the authors, without undue reservation.
